# Preoperative Risk Factors for Persistent Pain After Total Hip Arthroplasty for Hip Osteoarthritis: The Influence of Neuropathic Pain, Central Sensitization, and Pain Catastrophizing

**DOI:** 10.7759/cureus.80698

**Published:** 2025-03-17

**Authors:** Shinichi Ueki, Takeshi Shoji, Hiroki Kaneta, Hiroyuki Morita, Yosuke Kozuma, Nobuo Adachi

**Affiliations:** 1 Department of Orthopedic Surgery, Graduate School of Biomedical and Health Sciences, Hiroshima University, Hiroshima, JPN

**Keywords:** central sensitization, osteoarthritis of the hip, pain catastrophizing, persistent postoperative pain, total hip arthroplasty

## Abstract

Background

Approximately 10% of patients experience persistent pain after total hip arthroplasty (THA). Pain mechanisms, such as neuropathic pain, central sensitization, and pain rupture symptoms, have been reported to be associated with persistent postoperative pain. However, no studies have examined these mechanisms simultaneously. Therefore, in this study, we aimed to investigate the preoperative prevalence of neuropathic pain, central sensitization, and pain catastrophizing among patients with hip osteoarthritis (OA) and identify risk factors for persistent pain after THA, focusing on neuropathic pain, central sensitization, and pain catastrophizing.

Methods

In this retrospective study, 311 patients who underwent THA for hip OA were included. Preoperative neuropathic pain, central sensitization, and pain catastrophizing were evaluated using the pain-DETECT, Central Sensitization Inventory, and Pain Catastrophizing Scale, respectively. Persistent postoperative pain was defined as a Numerical Rating Scale score ≥3 at 12 months postoperatively. Persistent and non-persistent pain groups were compared using univariate and multivariate analyses.

Results

Preoperatively, neuropathic pain, central sensitization, and pain catastrophizing were present in 84 (27.0%), 74 (23.8%), and 183 (58.8%) of patients, respectively. In this study, 36 (11.6%) patients experienced persistent pain. The persistent pain group had a significantly higher prevalence of central sensitization and pain catastrophizing than the non-persistent pain group (*P *< 0.01). Multivariate analysis revealed central sensitization and pain catastrophizing as independent risk factors for persistent postoperative pain.

Conclusion

This study highlights the importance of preoperative assessment of central sensitization and pain catastrophizing in predicting persistent pain after THA for hip OA. Addressing these factors using targeted interventions may improve postoperative outcomes and patient satisfaction.

## Introduction

Osteoarthritis (OA) of the hip is a prevalent degenerative joint disease characterized by progressive cartilage loss, osteophyte formation, and joint space narrowing [1]. Total hip arthroplasty (THA) is a highly effective surgical intervention for hip OA, providing substantial relief from pain and improving function in most patients [2]. However, despite its overall success, approximately 10% of patients experience persistent postoperative pain, which is defined as pain that persists for ≥3 months postoperatively [3], affecting patients’ quality of life and causing major challenges to both patients and surgeons.

Understanding the mechanisms underlying persistent postoperative pain after THA is crucial to improving patient care and optimizing surgical techniques. The pain mechanism of hip OA is not only nociceptive pain but also neuropathic and nociplastic pain, including central sensitization and pain catastrophizing. Contributing factors, such as age, sex, obesity, neuropathic pain, central sensitization, and pain catastrophizing, have been identified in previous studies [4,5]. However, the existing literature is limited by comprehensive evaluations that consider all of these factors; therefore, the prevalence of neuropathic pain, central sensitization, and pain catastrophizing among patients who undergo THA for hip OA and the precise mechanisms underlying persistent pain after THA remain unknown.

In this study, we primarily aimed to investigate the preoperative prevalence of neuropathic pain, central sensitization, and pain catastrophizing among patients who underwent THA for hip OA and secondarily aimed to identify the risk factors for persistent pain after THA for hip OA, focusing on neuropathic pain, central sensitization, and pain catastrophizing. Overall, we aimed to elucidate the complex interplay among these factors and identify potential therapeutic targets and preventive measures to alleviate persistent postoperative pain and improve the outcomes of THA.

## Materials and methods

This study was approved by the local ethics committee of our institution, and informed consent was obtained from all participants.

Study population

**Figure 1:**
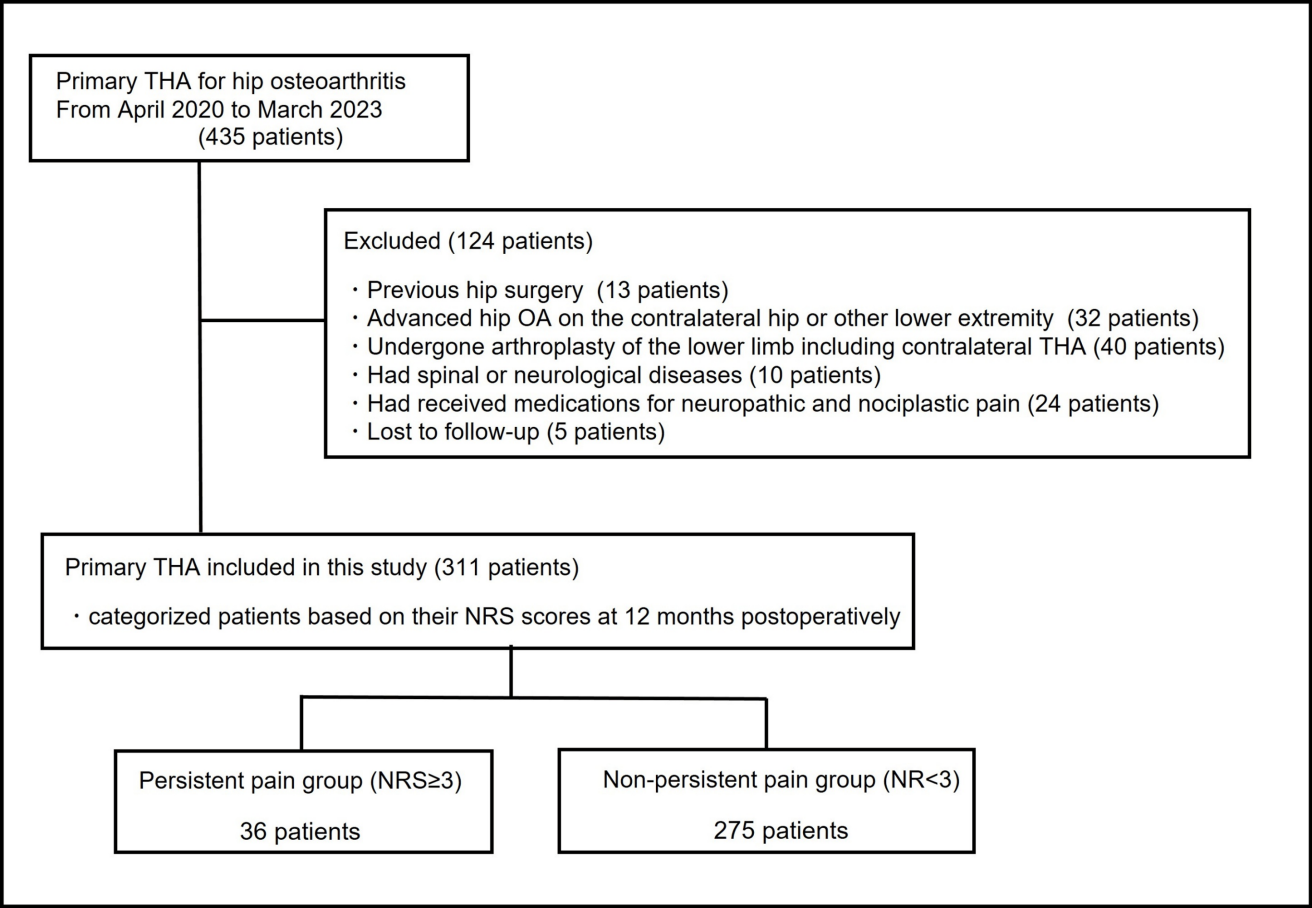
Flowchart of the distribution of all participants in the study. THA, total hip arthroplasty; NRS, Numerical Rating Scale

This study is a retrospective analysis conducted at a single institution. Between April 2020 and March 2023, 435 patients underwent primary THA (475 hips) for hip OA at our institution. According to radiographic findings, all hips were classified as Kellegren-Lawrence grade 3 or 4. Patients who had previously undergone hip surgery on the operative side, those who had end-stage OA on the contralateral hip or other lower extremity joints, those who had undergone arthroplasty of the lower limb including contralateral THA, those with spinal or neurological diseases, those who received medications for neuropathic and nociplastic pain, such as pregabalin, gabapentin, and duloxetine, and those who were lost to follow-up were excluded from this study. Finally, 311 patients, including 68 men and 243 women, with a mean age of 66.1 years, were included in this retrospective study (Figure 1). All THAs were conducted utilizing the same surgical assistance device and the anterolateral supine approach, performed by a single surgeon. All cup angles/positions were preoperatively planned using CT-based 3D templating and navigation software (CT-based Hip, version 1.0, Stryker Navigation, Freiburg, Germany). The cup was inserted with press-fit fixation with the assistance of the navigation system, and the stem was inserted under fluoroscopy for correct alignment and positioning. In the surgical procedure, either a cementless Taperloc microplasty stem and G7 acetabular cup (Zimmer Biomet Inc., Warsaw, IN) or an Accolade II femoral hip stem and Trident acetabular PSL hemispherical cup system (Stryker Orthopedics/Howmedica, Mahwah, NJ) was utilized, incorporating highly cross-linked polyethylene and a delta-ceramic head with a diameter of 32 or 36 mm. All patients received standardized postoperative therapies, including pain management and rehabilitation. Specifically, the duration of postoperative hospitalization generally ranged from 10 to 14 days. Ambulation, supported by the use of a walker or cane, was initiated on the first postoperative day under the supervision of a physical therapist. As inpatient pain management, a clinical pathway was implemented, involving the regular oral administration of celecoxib and the intermittent intravenous infusion of acetaminophen as needed. After discharge, patients were offered oral celecoxib based on their preferences.

Evaluation of preoperative pain mechanisms and persistent postoperative pain

One month prior to undergoing surgery, all patients completed validated questionnaires to evaluate their preoperative pain mechanisms. These questionnaires consisted of the Numerical Rating Scale (NRS) for assessing pain intensity, the pain-DETECT (PD) questionnaire for screening neuropathic pain, the Central Sensitization Inventory (CSI) for identifying central sensitization symptoms, and the Pain Catastrophizing Scale (PCS) for evaluating pain catastrophizing.

PD is a questionnaire used for screening neuropathic pain, consisting of nine items that assess pain quality, pattern, and radiation [6]. Possible scores range from 0 to 38, with higher scores indicating more neuropathic-like symptoms. We defined a score of ≥13 as indicative of neuropathic pain. Based on sensitivity, specificity, and predictive accuracy of 80-84% in a heterogeneous group of patients who experienced pain, neuropathic pain was screened using the following cut-off points relative to the clinical assessments of pain physicians: score ≤12 indicating unlikely neuropathic pain; score 13-18 indicating possibly neuropathic pain, and score ≥19 indicating likely neuropathic pain [6]. PD has been applied to several clinical populations, including those with knee OA and other musculoskeletal conditions, and the reported reliability and validity data have been generally favorable [7].

CSI was developed as a comprehensive screening tool for central sensitization. It incorporates Part A, which includes 25 statements pertaining to an individual's current health symptoms. Possible scores range from 0 to 100, with higher scores indicating more central sensitization, and the scores have been reported to have satisfactory reliability and consistency [8,9]. We defined a score of ≥40 as indicative of central sensitization based on previously reported cut-off values [8].

The PCS is a self-administered questionnaire for measuring catastrophic symptoms related to pain, and it was first reported in 1995 and subsequently translated into Japanese in 2006 [10,11]. The PCS measures catastrophic thinking and consists of three factors and 13 items, which are rated on a 5-point scale. Higher scores indicate a higher level of catastrophic thinking, and the PCS is a validated and commonly used instrument for measuring catastrophic thinking related to pain [12]. The association between PCS and persistent postoperative pain has been widely reported [13]. We defined a score of ≥30 as indicative of pain catastrophizing based on previously reported cut-off values [10]. Using these cut-off values, we first investigated the prevalence of neuropathic pain, central sensitization, and pain catastrophizing among patients who underwent THA for hip OA.

Furthermore, the level of postoperative pain in the operated hip was assessed using the NRS at 12 months postoperatively. An NRS score ≥3, corresponding to moderate or greater pain, was considered indicative of persistent postoperative pain [14]. We categorized patients into two groups based on their NRS scores at 12 months postoperatively: a persistent pain group (NRS score ≥3) and a non-persistent pain group (NRS score <3). Regarding the secondary objective of this study, a univariate analysis was conducted to compare the two groups based on age, gender, body mass index (BMI), preoperative NRS, neuropathic pain, central sensitization, and pain catastrophizing. Subsequently, a multivariate analysis was employed to identify the factors influencing postoperative persistent pain.

Data analysis

Statistical analyses were performed using EZR software (Saitama Medical Center, Jichi Medical University, Saitama, Japan), which is the graphical user interface for R (The R Foundation for Statistical Computing, Vienna, Austria). Statistical analysis was performed between the two groups using the Mann-Whitney U and Chi-square tests for univariate analysis. In addition, a logistic regression analysis was performed for multivariate analysis, using the explanatory variables that exhibited a value of <0.2 in the univariate analysis. Data were presented as mean (standard deviation), and statistical significance was set at P < 0.05.

## Results

No patient reported postoperative adverse events, such as hip dislocation, infection, fracture, or deep vein thrombosis. In this study, 36 (11.6%) patients experienced persistent pain at 12 months after THA for hip OA. There were no significant differences in patient demographics, including age, sex, BMI, and preoperative NRS score, between the persistent and non-persistent pain groups (Table 1).

**Table 1:** Patient demographics in each group BMI, body mass index; NRS, Numerical Rating Scale Data are expressed as mean (standard deviation)

Variables		Group		P-value
	Total	Persistent pain	Non-persistent pain	
Patients (n)	311	36	275	
Age (years)	66.1 (10.1)	66.3 (11.8)	66.1 (9.8)	0.54
Sex (male:female)	68:243	12:24	56:219	0.28
BMI (kg/m^2^)	23.7 (4.1)	24.4 (4.0)	23.6 (4.1)	0.16
Pre-operative NRS	7.8 (2.0)	8.1 (2.1)	7.8 (1.9)	0.21

Preoperatively, neuropathic pain, central sensitization, and pain catastrophizing were present in 84 (27.0%), 74 (23.8%), and 183 (58.8%) of patients, respectively, who underwent THA for hip OA. Of 36 patients in the persistent pain group, 13 (36.1%), 24 (66.7%), and 35 (97.2%) patients experienced preoperative neuropathic pain, central sensitization, and pain catastrophizing, respectively. Conversely, of 275 patients in the non-persistent postoperative pain group, 71 (25.8%), 50 (18.2%), and 148 (39.4%) patients experienced preoperative neuropathic pain, central sensitization, and pain catastrophizing, respectively. Univariate analysis (Table 2) revealed that the prevalence of central sensitization and pain catastrophizing was significantly higher in the persistent pain group (P < 0.01). Multivariate analysis, which included variables with a significance level of P < 0.2 in the univariate analysis, including BMI, neuropathic pain, central sensitization, and pain catastrophizing, was performed to ensure no multicollinearity issues. Logistic regression analysis revealed that central sensitization and pain catastrophizing were independently associated with persistent postoperative pain (Table 3).

**Table 2:** Univariate analysis of the occurrence of pain mechanisms between each group *P<0.05. **P<0.01.

Variables	Total	Group		P-value
		Persistent pain	Non-persistent pain	
Patients (n)	311	36 (11.6%)	275 (88.4%)	
Neuropathic pain (n)	84	13 (36.1%)	71 (25.8%)	0.09*
Central sensitization (n)	74	24 (66.7%)	50 (18.2%)	<0.01**
Pain catastrophizing (n)	183	35 (97.2%)	148 (39.4%)	<0.01**

**Table 3:** Multivariate logistic regression analysis of the influence of persistent postoperative pain BMI, body mass index; NRS, Numerical Rating Scale *P<0.01. **P<0.05.

Variables	Odds ratio (95% CI)	P-value
BMI (kg/m^2^)	1.0 (0.97-1.04)	0.78
Neuropathic pain	0.77 (0.28-2.09)	0.61
Central sensitization	6.76 (2.52-18.2)	<0.01*
Pain catastrophizing	13.7 (1.72-109)	0.01**

## Discussion

In this present study, we investigated the prevalence of preoperative pain mechanisms among patients who underwent THA for hip OA. Our findings demonstrated neuropathic pain in 84 (27.0%) patients, central sensitization in 74 (23.8%) patients, and pain catastrophizing in 183 (58.8%) patients out of a total of 311 patients. In addition, we identified preoperative risk factors for persistent postoperative pain, focusing on neuropathic pain, central sensitization, and pain catastrophizing. Univariate analysis revealed a higher prevalence of central sensitization and pain catastrophizing in the persistent postoperative pain group. Furthermore, the multivariate analysis revealed central sensitization and pain catastrophizing as independent factors associated with persistent postoperative pain.

The findings of this study are consistent with those of previous studies regarding the prevalence of neuropathic pain, central sensitization, and pain catastrophizing, as well as preoperative risk factors for persistent pain after THA. In previous studies, the rates of neuropathic pain, central sensitization, and pain catastrophizing before THA have been reported to be 24.5-32% [5,15], 9.9-24.6% [16,17], and 45% [18], respectively, which are comparable to the findings of the present study. In addition, factors such as preoperative central sensitization syndrome and pain catastrophizing have been associated with lower pain thresholds and persistent pain after THA [4,19]. To our knowledge, the present study is the first to investigate neuropathic pain, central sensitization, and pain catastrophizing simultaneously. Our findings support those of previous studies and provide insight into the complex relationship between these pain mechanisms. However, in this study, the presence of preoperative neuropathic pain did not influence persistent postoperative pain. Neuropathic pain is commonly associated with OA [20]; however, it has also been reported to improve after arthroplasty [5]. Neuropathic pain is considered to occur in the end stages of OA when the destruction of subchondral bone and inflammation of the synovium damage nerve endings in these areas [21]. Therefore, the elimination of stimulation to the subchondral bone and synovium after arthroplasty may be the reason for the improvement in postoperative neuropathic pain. However, chronic neuropathic pain has been reported to cause central sensitization, which can make treatment difficult [22]. In such cases, additional therapeutic intervention may be necessary. The observed association of preoperative central sensitization and pain catastrophizing with persistent postoperative pain suggests the involvement of complex pain mechanisms in the development of persistent pain after THA.

Pain catastrophizing has been proven to be a strong predictor of pain outcomes, including persistent postoperative pain and poor postoperative outcomes [13]. This mechanism involves fear of pain, which forms a vicious cycle of habitual pain avoidance behaviors, such as hyperalgesia, excessive rest, and shielding of the pain site. The involvement of catastrophizing has been proposed as a trigger for this fear [23]. Furthermore, pain catastrophizing, through cognitive and neurophysiological mechanisms, is a crucial psychological factor that may influence pain processing, treatment response, and, ultimately, the development of persistent postoperative pain.

Central sensitization is a condition that involves changes in the functioning of multiple interconnected components of the nervous system. It affects the facilitatory and inhibitory aspects of descending neurons, which regulate pain signals, and increases activity in several higher-level brain centers, including the anterior cingulate cortex, prefrontal cortex, and limbic system [24]. In addition, it has been reported that individuals who tend to focus excessively on pain sensations may experience heightened activation of their central neural mechanisms, leading to a persistent state of heightened sensitivity to pain. Notably, the relationship between pain catastrophizing and intensity has been reported to be mediated by central sensitization in previous studies [25,26], and the underlying complexity of central sensitization may reflect the associations between a larger pain extent and the potential presence of neuropathic pain and pain catastrophizing.

These findings underscore the significance of therapeutic interventions that target central sensitization. Duloxetine, a selective serotonin-norepinephrine reuptake inhibitor, has been reported to be effective in patients who have central sensitization and OA [27]. In a previous study, duloxetine was effective in preventing persistent pain after arthroplasty when administered during the perioperative period, suggesting that duloxetine may be effective in reducing pain and improving outcomes after arthroplasty in patients who have central sensitization [28]. However, evidence for the efficacy of duloxetine in patients who undergo THA is limited; therefore, further research is necessary to confirm its effectiveness.

This study contributes to the expanding body of evidence that emphasizes the significance of preoperative pain evaluation in predicting postoperative outcomes in patients who undergo THA. By identifying central sensitization and psychosocial pain as independent risk factors for persistent postoperative pain, healthcare providers can apply tailored interventions aimed at alleviating these factors preoperatively, thereby enhancing patient outcomes and satisfaction after THA.

This study has some limitations. First, its retrospective design restricted the establishment of causal relationships between preoperative pain mechanisms and persistent postoperative pain. In addition, the study population comprised patients who underwent THA for hip OA performed by a single surgeon at a single institution, which may affect the generalizability of the findings. However, this consistency in the study population helped to minimize confounding factors associated with surgical techniques and planning. Second, the presence of neuropathic pain, central sensitization, and pain catastrophizing was assessed using only questionnaires, and the cut-off values were established based on existing literature. However, few facilities possess equipment for quantitative sensory testing, which is used to assess central sensitization, and these questionnaires are widely available, making them highly versatile for screening risk factors for persistent postoperative pain. Additionally, because cut-off values for pain mechanisms have been reported to differ by race and disease [29,30], identifying optimal cut-off values for individual races and diseases is necessary in the future. Third, the accuracy of implant alignment after THA was not taken into consideration. However, THA was performed by one skilled orthopedic surgeon using CT-based 3D templating and navigation, thus minimizing major malalignment of implants. In addition to mechanical factors, non-mechanical factors such as preoperative hip deformity, soft tissue tension, and individual anatomical variation may influence postoperative outcomes. These factors could contribute to altered load distribution and joint kinematics, potentially affecting pain and functional recovery. Therefore, further research is needed to integrate these factors. Fourth, the effects of pharmacological after post-discharge treatment have not been considered. Patients who were administered medications for neuropathic and nociceptive pain prior to surgery were excluded from the study, and during their hospitalization, pain management was standardized according to the clinical pathway. However, the specific analgesics utilized at other hospitals following discharge remain unclear. Future research should involve a prospective study with fully standardized pain management protocols.

## Conclusions

Preoperative assessment of pain mechanisms, including central sensitization and pain catastrophizing, is crucial to identifying patients at risk of persistent pain after THA for hip OA. Addressing these mechanisms using targeted interventions may improve postoperative outcomes and patient satisfaction. Future research should focus on determining the underlying mechanisms and developing personalized strategies for pain management to optimize outcomes in patients who undergo THA.
